# Identification of key lncRNAs in the tumorigenesis of intraductal pancreatic mucinous neoplasm by coexpression network analysis

**DOI:** 10.1002/cam4.2927

**Published:** 2020-04-02

**Authors:** Jun Ding, Yi Li, Yong Zhang, Bin Fan, Qinghe Li, Jian Zhang, Jiayao Zhang

**Affiliations:** ^1^ Department of Hepatobiliary Surgery The Central Hospital of Enshi Autonomous Prefecture Enshi China

**Keywords:** coexpression network analysis, intraductal papillary mucinous neoplasm, lncRNA, tumorigenesis

## Abstract

Intraductal papillary mucinous neoplasm (IPMN) is an intraepithelial precancerous lesion of pancreatic ductal adenocarcinoma (PDAC) that progresses from adenoma to carcinoma, and long noncoding RNAs (lncRNA) might be involved in the tumorigenesis. In this study, we obtained the expression profiles of more than 4000 lncRNAs by probe reannotation of a microarray dataset. As a correlation network‐based systems biology method, weighted gene coexpression network analysis (WGCNA) was used to find clusters of highly correlated lncRNAs in the tumorigenesis of IPMN, which covered four stepwise stages from normal main pancreatic duct to invasive IPMN. In the most relevant module (R^2^ = −0.75 and *P* = 5E‐05), three hub lncRNAs were identified (HAND2‐AS1, CTD‐2033D15.2, and lncRNA‐TFG). HAND2‐AS1 and CTD‐2033D15.2 were negatively correlated with the tumorigenesis (*P* in one‐way ANOVA test = 1.45E‐07 and 1.39E‐0.5), while lncRNA‐TFG were positively correlated with the tumorigenesis (*P* = 3.99E‐08). The validation set reached consistent results (*P* = 2.66E‐03 in HAND2‐AS1, 1.47E‐04 in CTD‐2033D15.2 and 6.23E‐08 in lncRNA‐TFG). In functional enrichment analysis, the target genes of microRNAs targeting also these lncRNAs were overlapped in multiple biological processes, pathways and malignant diseases including pancreatic cancer. In survival analysis, patients with higher expression of HAND2‐AS1‐targeted and CTD‐2033D15.2‐targeted microRNAs showed a significantly poorer prognosis in PDAC, while high expression of lncRNA‐TFG‐targeted microRNAs demonstrated an obviously better prognosis (log‐rank *P* < .05). In conclusion, by coexpression network analysis of the lncRNA profiles, three key lncRNAs were identified in association with the tumorigenesis of IPMN, and those lncRNAs might act as early diagnostic biomarkers or therapeutic targets in pancreatic cancer.

## Graphical abstract

Intraductal papillary mucinous neoplasm (IPMN) is an intraepithelial precancerous lesion of pancreatic ductal adenocarcinoma (PDAC) that progresses from adenoma to carcinoma, and long noncoding RNAs (lncRNA) might be involved in the tumorigenesis. In this study, we obtained the expression profiles of more than 4000 lncRNAs by probe reannotation of a microarray dataset. As a correlation network‐based systems biology method, weighted gene coexpression network analysis (WGCNA) was used to find clusters of highly correlated lncRNAs in the tumorigenesis of IPMN, which covered four stepwise stages from normal main pancreatic duct to invasive IPMN. Magenta module was identified as the most relevant module.

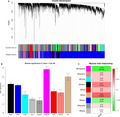

## 1 INTRODUCTION

Pancreatic cancer is one of the common cancers across the world, characterized by a poor prognosis.^1^ The dominant subtype of pancreatic ductal adenocarcinoma (PDAC) accounts for more than 90%.^2^ As an intraepithelial precancerous lesion of pancreatic cancer, intraductal papillary mucinous neoplasm (IPMN) could progress from adenoma to PDAC. Several factors have been identified in relation with the tumorigenesis of IPMN, like blood type, main duct dilatation, and high‐grade dysplasia, but the mechanism is still unclear.^3^, ^4^, ^5^ As a diverse class of transcribed RNA molecules consisting of more than 200 nucleotides, long noncoding RNAs (lncRNAs) played an important role in the regulation of protein‐coding gene expression, although they did not encode proteins.^6^, ^7^ Recent studies also reported a regulatory role of lncRNAs in the proliferation, invasion, and metastasis of pancreatic cancer. For example, lncRNA TUSC7, LINC00052, and lncRNA CASC2 modulated miR‐371a‐5p, miR‐330‐3p, and miR‐21, respectively, to suppress pancreatic cancer cell lines.^8^, ^9^, ^10^ High expression of lncRNA MALAT1 was an independent predictor for overall survival in PDAC.^11^ However, few studies investigated the role of lncRNAs in IPMN. In the study of Permuth et al, the signature of eight circulating lncRNAs were more accurate than clinical features in the differential diagnosis of malignant and benign IPMN.^12^

High‐throughput microarray technology develops rapidly in recent years, and several studies adopted the gene expression profiles to find genes associated with the tumorigenesis and prognosis of pancreatic cancer. Nevertheless, most studies focused on the differentially expressed protein‐coding genes in pancreatic cancer, regardless of the vast majority of lncRNAs and the intermediate stages, like IPMN. As a correlation network‐based systems biology method, weighted gene coexpression network analysis (WGCNA) was used to find clusters of highly related genes and clusters to clinical features.^13^ In this study, as the lncRNA expression profiles were unavailable, we obtained the lncRNA expression data through an accurate reannotation of the microarray probe.^13^ Then, the WGCNA method was used to find network hub lncRNAs related with the tumorigenesis of IPMN.

## 2 MATERIALS AND METHODS

### 2.1 Data collection

We obtained the raw data of gene expression profiles (CEL files) and clinical data of dataset GSE19650 and GSE26647 from the database of Gene Expression Omnibus (GEO) (http://www.ncbi.nlm.nih.gov/geo/).^14^, ^15^ Dataset GSE19650 was conducted on the platform of GPL570 (Affymetrix Human Genome U133 Plus 2.0 Array), and used as a training set to construct the coexpression network and identify hub genes in this study. It had 22 tissue samples which were sufficient for the subsequent WGCNA analysis, and covered four sequential stages, including normal main pancreatic duct (n = 7), intraductal papillary mucinous adenoma (IPMA) (or low‐grade dysplasia in IPMN) (n = 6), intraductal papillary mucinous carcinoma (IPMC) (or high‐grade dysplasia in IPMN) (n = 6), and invasive IPMN (n = 3). All patients undergone initial surgical resection, and received no prior therapy. The tumors were classified according to the combined criteria.^14^, ^16^ Another dataset GSE26647 was conducted on the platform of GPL5175 (Affymetrix Human Exon 1.0 ST), and used as a validation set to verify the results. It had 28 tissue samples, and covered four sequential stages of IPMN, including low‐grade (n = 10), moderate‐grade (n = 5), high‐grade (n = 6), and invasive IPMN (n = 7).

### 2.2 Data preprocessing

**Figure 1:**
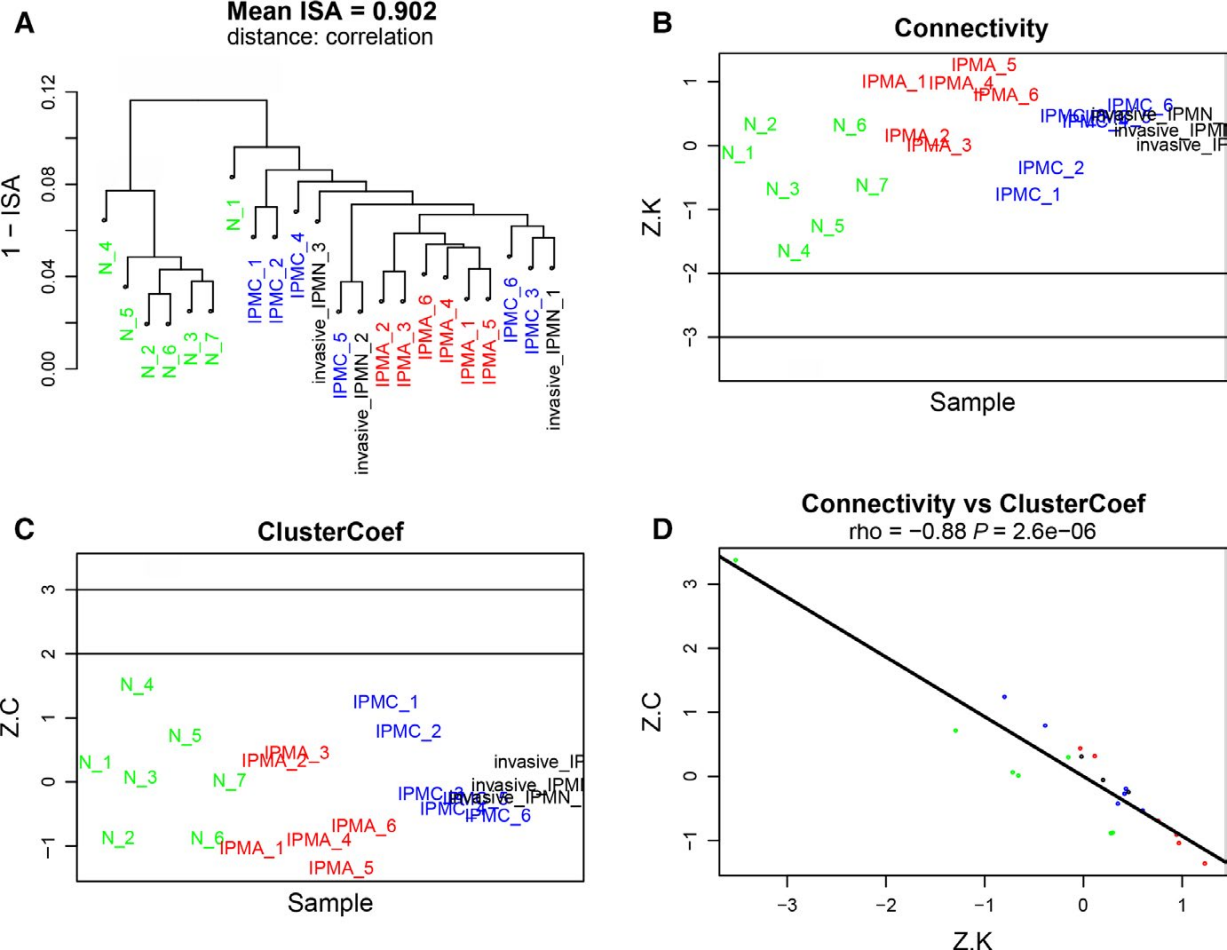
Sample network concepts to detect outliers in the training set. A, Dendrogram produced by average linkage hierarchical clustering using 1‐ISA (intersample adjacency). B, Standardized sample connectivities (Z.K) for the samples. C, Standardized sample clustering coefficients (Z.C) for the samples. D, Correlation between standardized sample connectivities (Z.K) and standardized sample clustering coefficients (Z.C) for the samples

The R software package of “affy” was used to preprocess the raw expression data according to the RMA method: (a) background correction; (b) log2 transformation; (c) quantile normalization; (d) median polish summarization. In quality assessment, sample outliers were removed using the network method for describing sample relationships in genomic datasets, with the threshold of standardized sample connectivity (Z.K)>−2 and standardized sample clustering coefficient (Z.C) <2.^17^ Finally, we found no outliers in both the datasets (Figure 1A‐D).

### 2.3 Probe reannotation

As for the GPL570 microarray, the fasta file of probe sequences was obtained from the annotation file (http://www.affymetrix.com). In the GENCODE database, we downloaded the fasta file of human genome (GRCh38) and the gtf file of annotation file (GRCh38.p12) (https://www.gencodegenes.org). Probe‐matched lncRNA sequences were identified by the spliced alignment program for reads alignment of HISAT.^18^ In this study, we defined the lncRNA transcripts as those with the gene types of "lincRNA", "bidirectional_promoter_lncRNA", "macro_lncRNA", "antisense", "processed_transcript", "TEC", "3prime_overlapping_ncRNA", "sense_intronic", "non_coding", and "sense_overlapping". We included the transcripts according to the following criteria: (a) identified by ≥4 probes; (b) perfect match; (c) specific match.^13^ If multiple probes mapped to the same lncRNA, we assigned the mean value to the lncRNA. As for the GPL5175 microarray, the probes were reannotated using the same method and criteria.

### 2.4 Coexpression network construction

**Figure 2:**
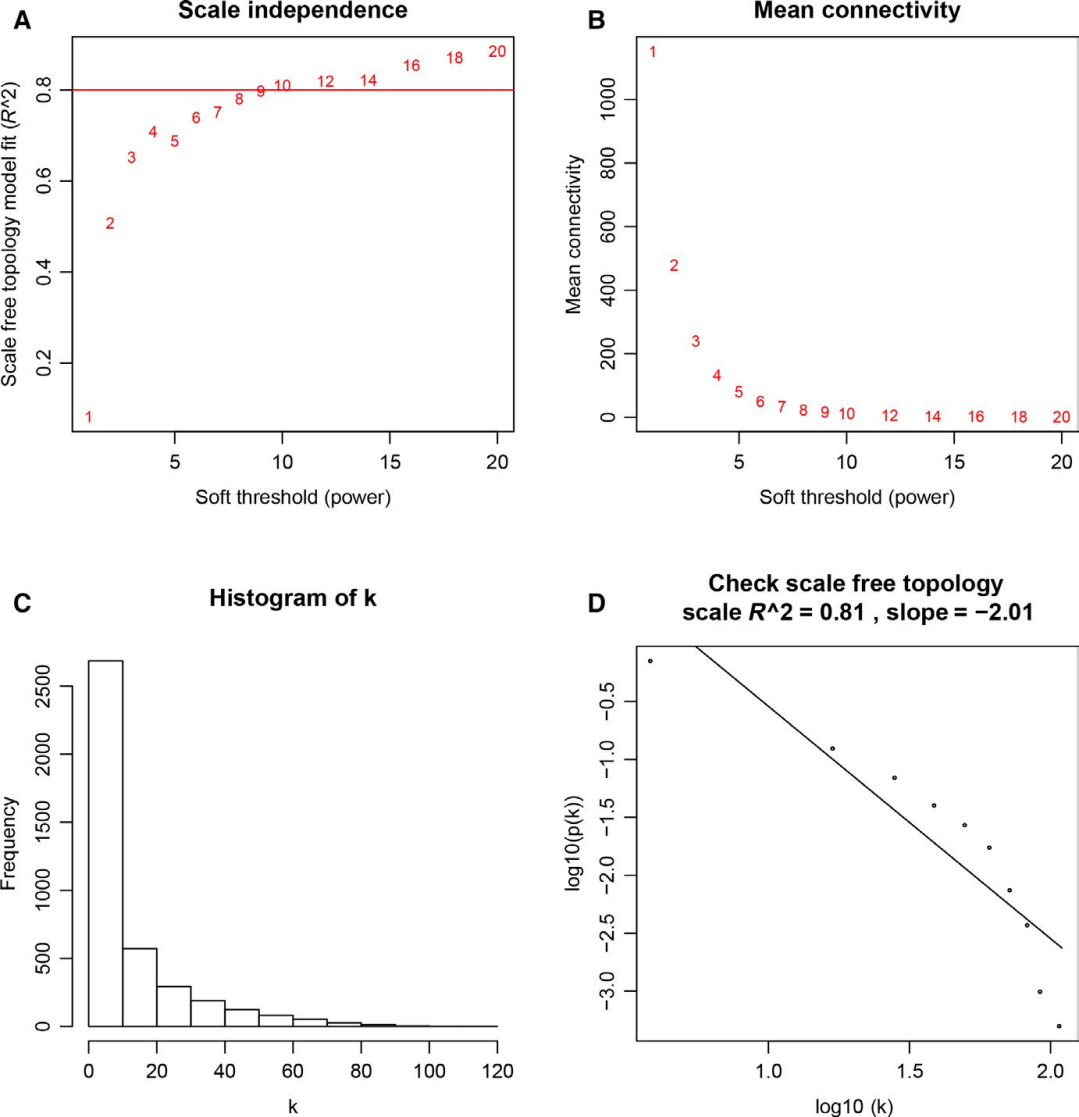
Determination of soft‐thresholding power in the coexpression network analysis. A, Analysis of the scale‐free fit index for various soft‐thresholding powers (β). B, Analysis of the mean connectivity for various soft‐thresholding powers. C, Histogram of connectivity distribution when β = 9. D, Checking the scale free topology when β = 9

The Pearson's distances of each paired lncRNAs were calculated by the construction of a weighted adjacency matrix, in which we chose a power of 9 to ensure a scale‐free network (scale free R^2^ = 0.81) (Figure 2A‐D).^19^ In the network, the hubs were defined as the highest‐degree nodes, which usually played certain roles. Then, we transformed the adjacency matrix into the topological overlap matrix (TOM). In TOM, the network connectivity of a lncRNA could be measured by calculating the sum of its adjacency with all other lncRNAs for network generation.^20^ Then, average linkage hierarchical clustering were used to divide the lncRNAs with similar expression patterns in the same modules (a minimum size of at least 30).^21^ The analysis was conducted by the R software package of “WGCNA”, and the R script used in this study was available on the tutorial website (https://horvath.genetics.ucla.edu/html/CoexpressionNetwork/Rpackages/WGCNA/Tutorials).

### 2.5 Significant module identification

The module in relation to the IPMN tumorigenesis was identified by two methods.^13^ The significant module was defined as the module with the module with the maximal absolute module significance (MS) which was the average lncRNA significance in the module. Second, we also calculated the correlation between the first principal component in the principal component analysis for each module and the clinical features to find the most correlative module.

### 2.6 Identification of hub lncRNAs

As the highly interconnected nodes in a module, hub genes in the coexpression network have been functionally reported.^22^ In this study, we calculated the intramodular connectivity of each lncRNA and high module membership (MM) by the Pearson's correlation. Then, we chose the lncRNAs with both high intramodular connectivity and high MM as the hub genes (namely cor.Standard >0.8 and cor.Weighted >0.8).^13^

### 2.7 Functional enrichment analyses

The cDNA sequences of hub lncRNAs were obtained from the database of Ensembl (http://asia.ensembl.org). The microRNA targets of these hub lncRNAs were predicted by blasting the sequence with the microRNAs (http://mirdb.org/miRDB/custom.html). The gene targets of the microRNAs were obtained from the experimental validation database of miRTarBase (http://mirtarbase.mbc.nctu.edu.tw/php/index.php), and two computational prediction databases of miRDB (http://mirdb.org/) and TargetScan (http://www.targetscan.org/). To study the potential function of these microRNAs, we performed a gene ontology (GO) analysis of related biological processes, pathways and diseases on their predictive gene targets using the online tool of ToppFun (https://toppgene.cchmc.org/enrichment.jsp). A false discovery rate (FDR) of less than 0.05 was selected as the cut‐off.

### 2.8 Survival analyses

As the survival data of novel lncRNAs were unavailable, the prognostic role of these lncRNAs in PDAC was indirectly evaluated by investigating the association between their target microRNAs and the prognosis. Kaplan‐Meier plotter (http://kmplot.com) was an online tool based on the RNA‐sequencing data and clinical data of The Cancer Genome Atlas (TCGA) dataset, and was used to study the relationship between the microRNA expression and PDAC prognosis.

## 3 RESULTS

### 3.1 Network construction and significant module identification

**Figure 3:**
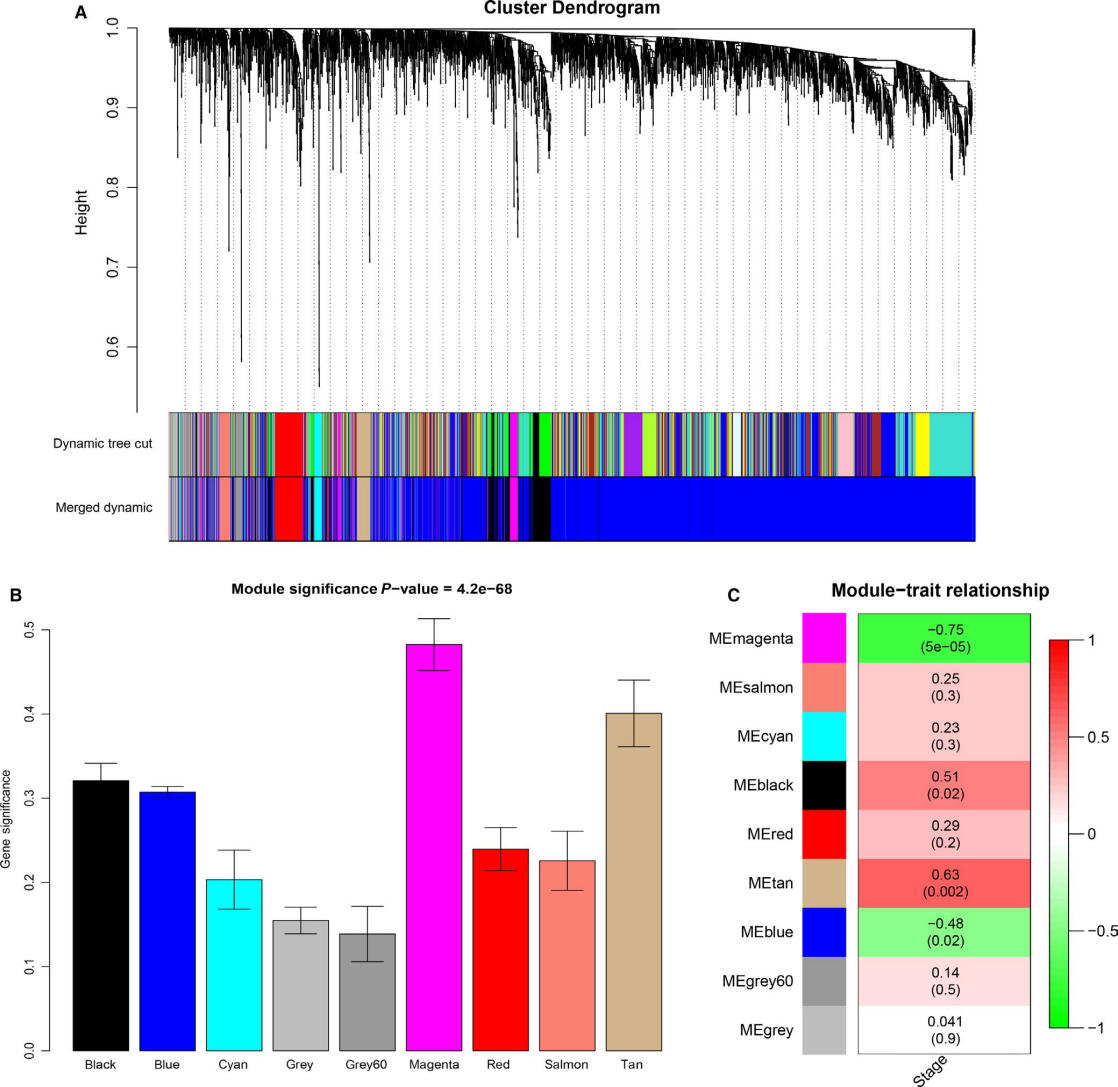
Identification of modules associated with the tumorigenesis of IPMN. A, Dendrogram of 4021 lncRNAs clustered based on a dissimilarity measure (1‐TOM). B, Distribution of average gene significance in the modules associated with the tumorigenesis. C, Heatmap of the correlation between module eigengenes and the tumorigenesis

In the training set, probe annotation identified a total of 4951 probes which were mapped to a total of 4066 lncRNAs. According to the similarity of expression pattern, these lncRNAs were grouped into nine modules (black (lncRNA number: 362), blue (2957), cyan (66), grey (195), grey60 (41), magenta (124), red (155), salmon (71), tan (95)) (Figure 3A). Additionally, the grey module was the module of lncRNAs not assigned to any module, which was regarded as a special module and not considered. In the magenta module, there shared the highest MS, and the ME also had a higher correlation than other modules in the tumorigenesis (R^2^ = −0.75 and *P* = 5E‐05) (Figure 3B,C). Two methods reached the consistent result, and thus the magenta module was chose as the relevant module in the tumorigenesis of IPMN.

### 3.2 Identification and validation of hub lncRNAs

**Figure 4:**
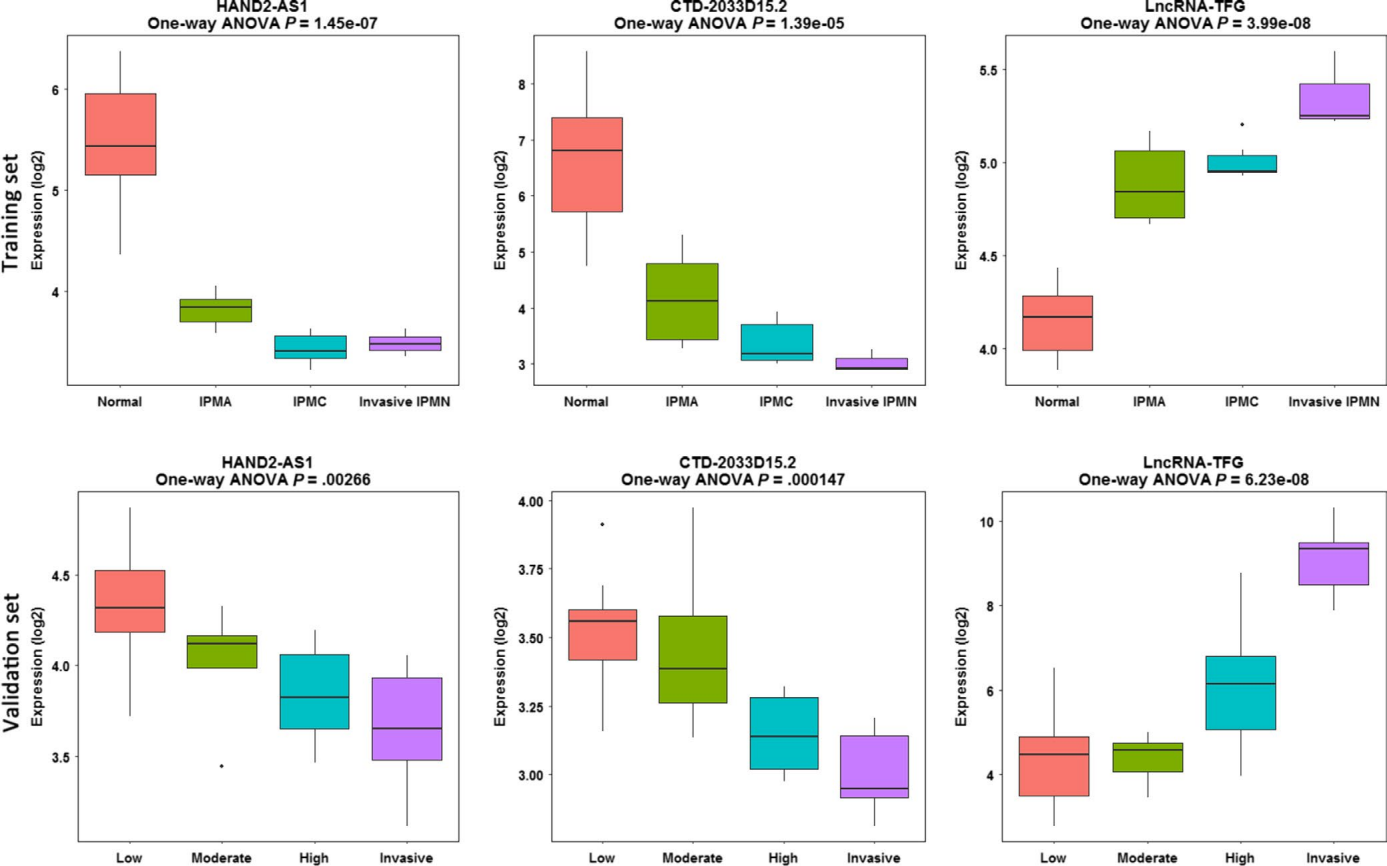
Boxplot of the hub lncRNAs across four stepwise stages in the tumorigenesis of IPMN. IPMA, intraductal papillary mucinous adenoma; IPMC, intraductal papillary mucinous carcinoma; IPMN, intraductal papillary mucinous neoplasm; low, low‐grade IPMN; moderate, moderate‐grade IPMN; high, high‐grade IPMN; invasive, invasive IPMN

In the magenta module, hub lncRNAs were defined as the lncRNAs with both high intra‐modular connectivity (cor.Standard > 0.8) and high MM (cor.Weighted > 0.8) (Table 1). Finally, three lncRNAs were identified, namely the antisense of HAND2‐AS1, the sense intronic of CTD‐2033D15.2 and the processed transcript of lncRNA‐TFG. HAND2‐AS1 and CTD‐2033D15.2 were negatively correlated with the tumorigenesis (*P* in one‐way ANOVA test = 1.45E‐07 and 1.39E‐0.5), while lncRNA‐TFG were positively correlated with the tumorigenesis (*P* = 3.99E‐08) (Figure 4). In the validation set, HAND2‐AS1 and CTD‐2033D15.2 were in negative correlation with the four stepwise stages of IPMN (*P* in one‐way ANOVA test = 2.66E‐03 and 1.47E‐04), and lncRNA‐TFG in positive correlation with the progression (*P* = 6.23E‐08). The results indicated a protective role of HAND2‐AS1 and CTD‐2033D15.2, and a risk factor of lncRNA‐TFG in the tumorigenesis of IPMN.

**Table 1:** Hub lncRNAs in the magenta module

Gene symbol	LncRNA type	Ensembl ID	Probe ID	Coexpression analysis			
				p.Weighted	p.Standard	cor.Weighted	cor.Standard
HAND2‐AS1	Antisense	ENSG00000237125 (ENST00000561655.2)	219791_s_at, 236141_at, 239708_at	2.00E‐11	6.04E‐06	−0.948	−0.806
CTD‐2033D15.2	Sense_intronic	ENSG00000276107 (ENST00000478845.2)	239336_at	3.49E‐10	7.16E‐06	−0.931	−0.802
LncRNA‐TFG	Processed_transcript	ENSG00000114354 (ENST00000481203.1)	239385_at, 244614_at	4.47E‐11	6.10E‐08	0.944	0.881

### 3.3 Functional enrichment analyses

**Figure 5:**
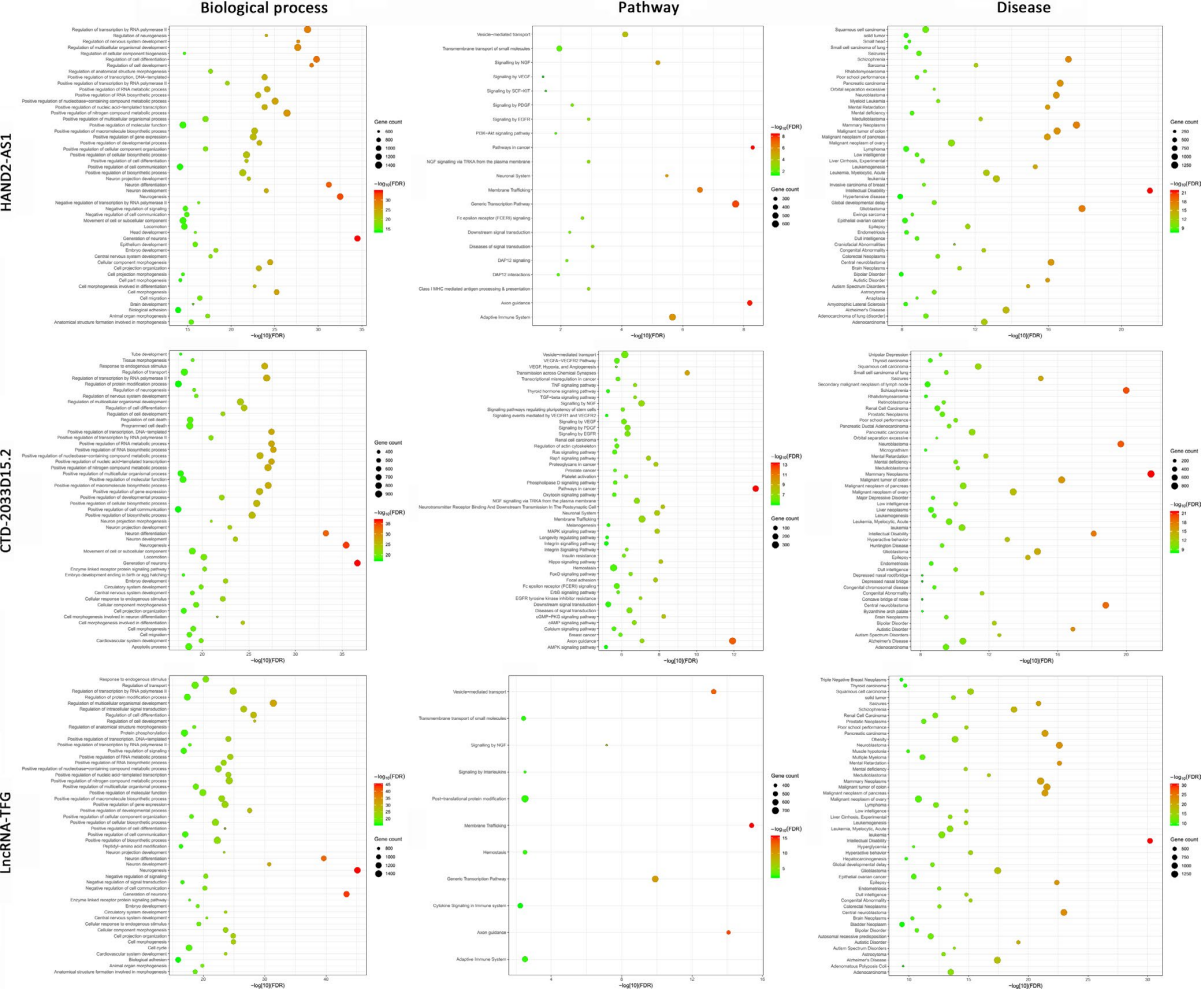
Functional enrichment analyses (biological process, pathway, and disease) of the target genes of microRNAs targeting also the hub lncRNAs

Fifty‐four microRNAs were identified as the targets of HAND2‐AS1, while 15 microRNAs were in CTD‐2033D15.2 and 56 microRNAs in lncRNA‐TFG. Multiple biological processes, pathways and diseases were, respectively, enriched in the targets genes of microRNAs targeting also these lncRNAs (Figure 5). In the overlap analysis, the target genes have the common biological processes of cell differentiation and development especially for neuron, the pathways of axon guidance, membrane trafficking, vesicle‐mediated transport and signaling by NGF, the diseases of mental disorders, and malignant diseases including pancreatic cancer. The results indicated a potential involvement of these lncRNAs in the tumorigenesis of PDAC, including the intermediate stage of IPMN.

### 3.4 Expression levels of lncRNA‐targeted microRNAs and PDAC prognosis

**Figure 6:**
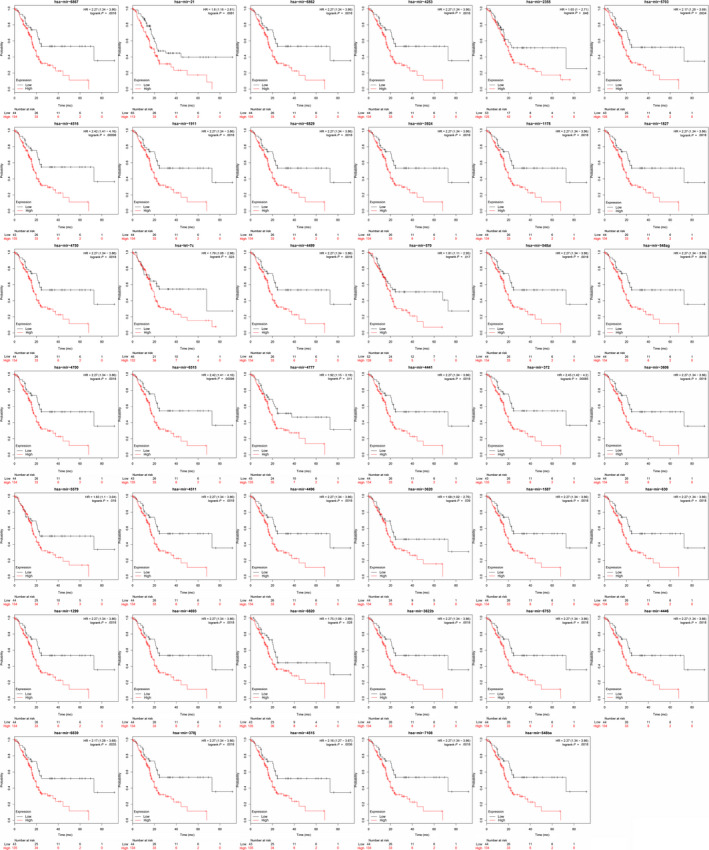
Expression levels of the HAND2‐AS1‐targeted microRNAs and the prognosis of pancreatic ductal adenocarcinoma (PDAC)

**Figure 7:**
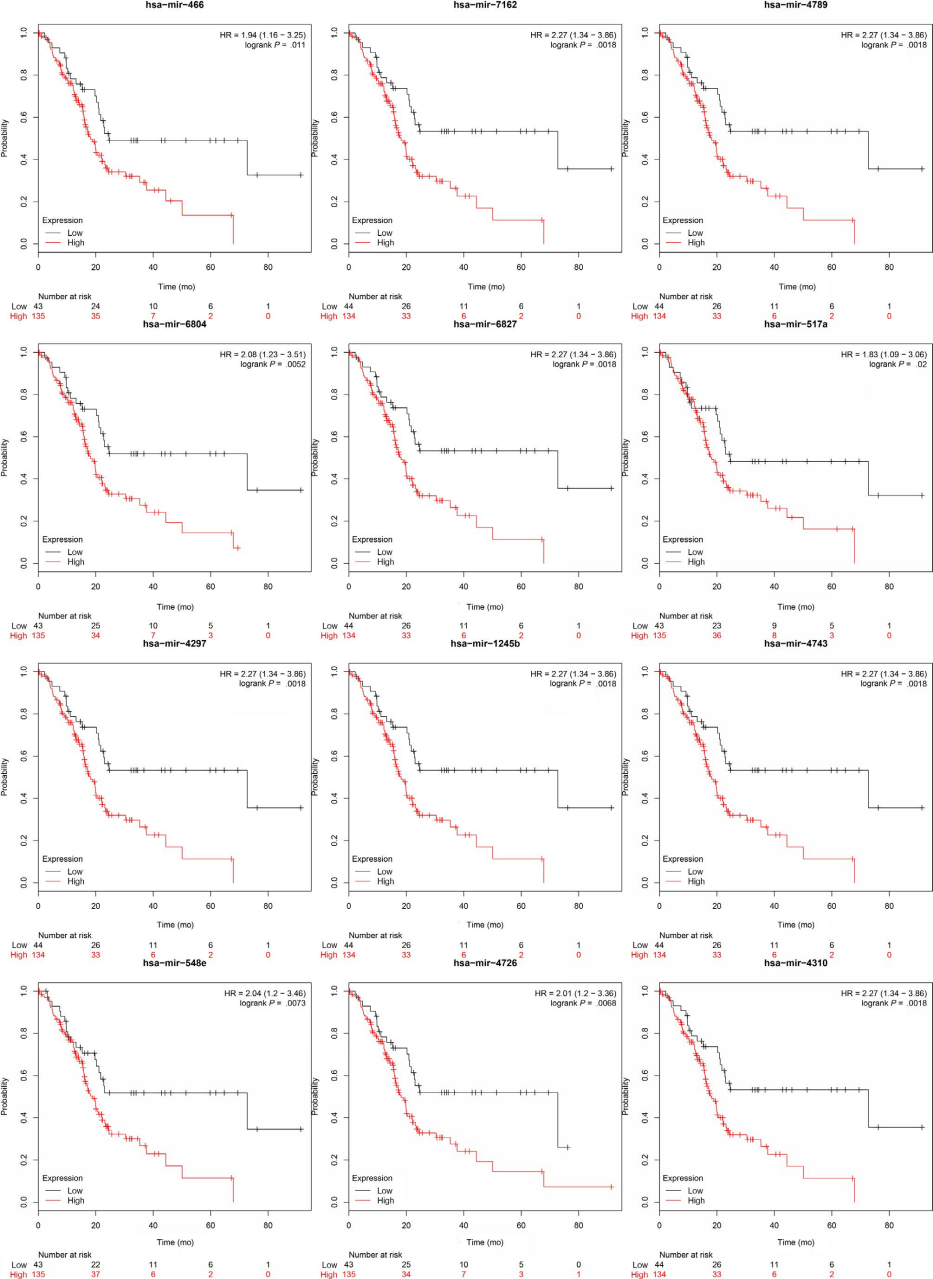
Expression levels of the CTD‐2033D15.2‐targeted microRNAs and the prognosis of pancreatic ductal adenocarcinoma (PDAC)

**Figure 8:**
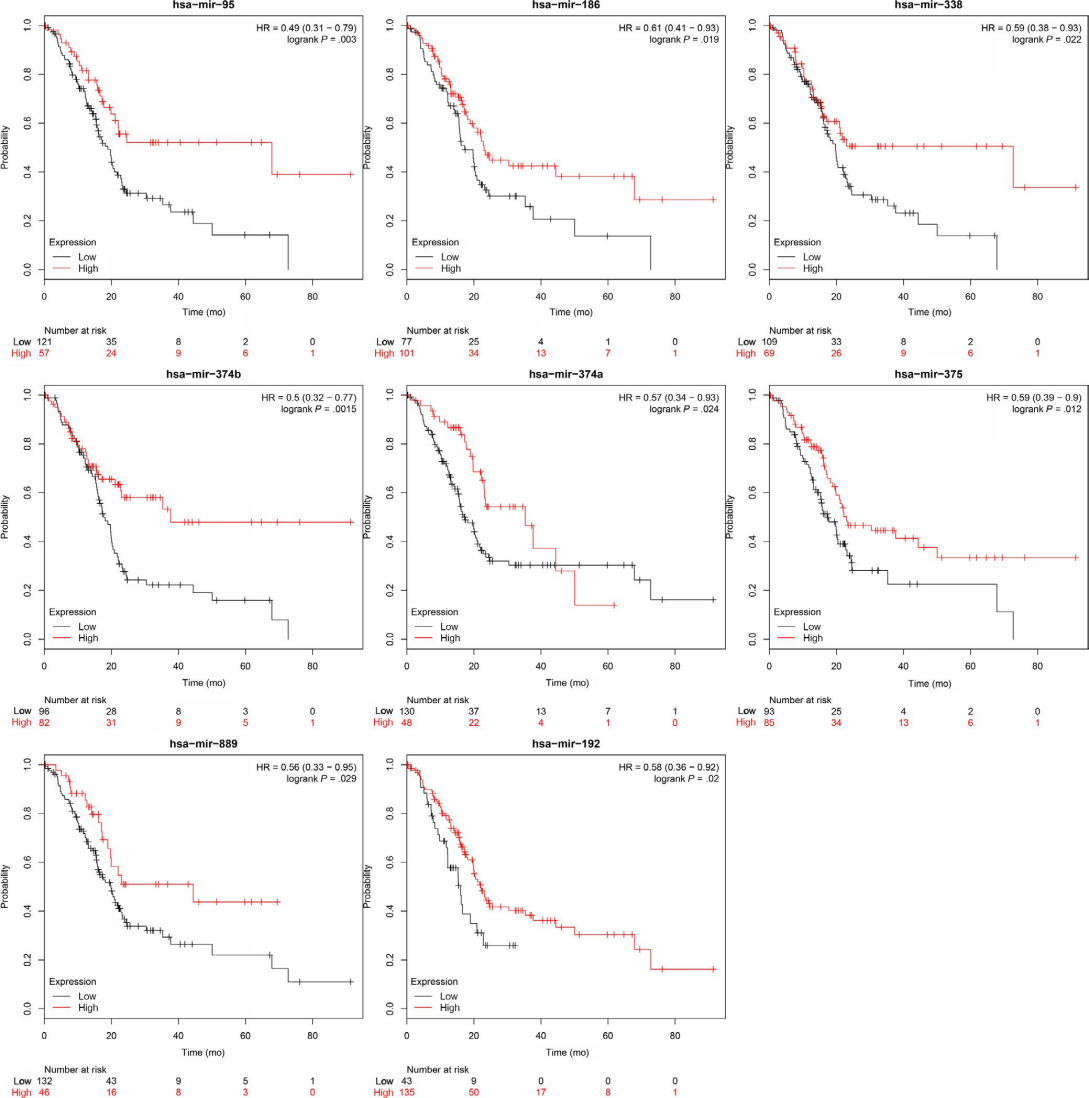
Expression levels of the lncRNA‐TFG‐targeted microRNAs and the prognosis of pancreatic ductal adenocarcinoma (PDAC)

Among 41 (76%) of the 54 HAND2‐AS1‐targeted microRNAs and 13 (87%) of the 15 CTD‐2033D15.2‐targeted microRNAs, patients with higher microRNA expression showed a significantly poorer prognosis in PDAC (log‐rank *P* < .05) (Figures 6 and 7). LncRNA‐TFG was the processed transcript of protein‐coding gene TFG, and 11 specific microRNAs were identified from the 56 lncRNA‐TFG‐targeted microRNAs when compared with TFG mRNA. Among 8 (73%) of the 11 microRNAs, patients with higher microRNA expression showed a significantly better prognosis (Figure 8). The results indicated a protective role of HAND2‐AS1 and CTD‐2033D15.2, and a risk factor of lncRNA‐TFG in PDAC.

## 4 DISCUSSION

Initially, lncRNAs were thought to be nonfunctional in transcription, but recent studies experimentally validated that multiple lncRNAs could sponge microRNAs to up‐regulate downstream message RNAs.^23^ Recently, several studies have reported the regulatory role of lncRNAs in the etiology of pancreatic cancer. Dysregulated expression of multiple lncRNAs has been documented in pancreatic cancer, which might be a major cause of tumorigenesis and progression.^24^ Furthermore, epigenetic events were proposed to have an important role in the tumorigenesis of pancreatic cancer, while lncRNAs has already been found as important epigenetic regulators in multiple biological processes, such as cell proliferation, differentiation and apoptosis and subsequent tumorigenesis.^25^ Several lncRNAs have been experimentally validated in the tumorigenesis and progression of pancreatic cancer. For example, certain lncRNAs could interact with the WDR5/MLL complex to up‐regulate the expression of multiple 5’ HOXA genes, which played an regulatory role in the progression and chemoresistance of pancreatic cancer.^26^

The study of Giulietti et al^27^ also used the method of coexpression network analysis to identify novel lncRNA biomarkers for PDAC. Finally, 11 lncRNAs were found, namely A2M‐AS1, DLEU2, MIR155HG, SLC25A25‐AS1, ITGB2‐AS1, TSPOAP1‐AS1, LINC01133, LINC00675, PSMB8‐AS1, LINC01857, and LOC642852. However, Giulietti *et al* extracted the lncRNAs by the microarray annotation file, which was different from the method of probe reannotation. Fu et al^28^ study adopted the same method as us and identified three lncRNAs (AFAP1‐AS1, UCA1, and ENSG00000218510) involved in the PDAC progression. However, few studies performed a systematic analysis to find key lncRNAs in relation to the tumorigenesis of IPMN.

In this study, we combined the method of probe reannotation with coexpression network analysis, and identified three lncRNAs (HAND2‐AS1, CTD‐2033D15.2, and lncRNA‐TFG) in association with the tumorigenesis of IPMN. In functional enrichment analysis, the target genes of microRNAs targeting also these lncRNAs were enriched in multiple biological processes, pathways, and malignant diseases including pancreatic carcinoma. In survival analysis, the target microRNAs of the lncRNAs were also correlated with the prognosis of PDAC. These results suggested a potential role of these lncRNAs in the pancreatic tumorigenesis. In mechanism, these lncRNAs might act though sponging the target microRNAs which were involved in the pathogenesis of pancreatic malignancies. Among these lncRNAs, HAND2‐AS1 has been experimentally validated as a suppressor in multiple cancers, like esophagus squamous cell cancer, nonsmall cell lung cancer, colorectal cancer, osteosarcoma, and endometrioid endometrial cancer.^29^, ^30^, ^31^, ^32^, ^33^

As far as we know, this was the first study that combined the method of probe reannotation with coexpression network analysis to find key lncRNAs in relation to the tumorigenesis of IPMN. However, the limitations should be also acknowledged. First, the method of probe reannotation could help obtain reliable lncRNA data, but not cover all lncRNAs. Second, cellular and molecular experiments were needed to validate our results.

In conclusion, by coexpression network analysis of the lncRNA profiles, three key lncRNAs were identified in association with the tumorigenesis of IPMN, and those lncRNAs might act as early diagnostic biomarkers or therapeutic targets in pancreatic cancer.

## CONFLICT OF INTERESTS

None.

## AUTHOR CONTRIBUTIONS

Jun Ding, Yi Li, and Jiayao Zhang participated in the development of the study concept, performed data analysis and interpretation of the data and participated in the writing of the manuscript. Yong Zhang participated in the development of the study concept, provided data for the study, performed data analysis and interpretation of the data and participated in the writing of the manuscript. Bin Fan participated in the development of the study concept, interpretation of the data and in the writing of the manuscript. Qinghe Li participated in the interpretation of the data, and in the writing of the manuscript. Jian Zhang provided data for the study.

## Data Availability

Data available on request from the authors.

## References

[1] Weaver DT , Lietz AP , Mercaldo SF , et al. Testing for verification bias in reported malignancy risks for side‐branch intraductal papillary mucinous neoplasms. A simulation modeling approach. Am J Roentgenol. 2019;212(3):596‐601. 30620679 10.2214/AJR.18.20180 PMC8576744

[2] Tempero MA , Malafa MP , Al‐Hawary M , et al. Pancreatic adenocarcinoma, version 2.2017, NCCN clinical practice guidelines in oncology. J Natl Compr Canc Netw. 2017;15(8):1028‐1061. 28784865 10.6004/jnccn.2017.0131

[3] Del Chiaro M , Beckman R , Ateeb Z , et al. Main duct dilatation is the best predictor of high‐grade dysplasia or invasion in intraductal papillary mucinous neoplasms of the pancreas. Ann Surg. 2019. 10.1097/SLA.0000000000003174 30672797

[4] Poruk KE , Griffin J , Makary MA , et al. Blood type as a predictor of high‐grade dysplasia and associated malignancy in patients with intraductal papillary mucinous neoplasms. J Gastrointest Surg. 2019;23(3):477‐483. 30187322 10.1007/s11605-018-3795-9 PMC6399082

[5] Rezaee N , Barbon C , Zaki A , et al. Intraductal papillary mucinous neoplasm (IPMN) with high‐grade dysplasia is a risk factor for the subsequent development of pancreatic ductal adenocarcinoma. HPB (Oxford). 2016;18(3):236‐246. 27017163 10.1016/j.hpb.2015.10.010 PMC4814593

[6] Marchese FP , Raimondi I , Huarte M . The multidimensional mechanisms of long noncoding RNA function. Genome Biol. 2017;18(1):206. 29084573 10.1186/s13059-017-1348-2 PMC5663108

[7] Long Y , Wang X , Youmans DT , Cech TR . How do lncRNAs regulate transcription? Sci Adv. 2017;3(9):eaao2110. 28959731 10.1126/sciadv.aao2110 PMC5617379

[8] Yue L , Guo J . LncRNA TUSC7 suppresses pancreatic carcinoma progression by modulating miR‐371a‐5p expression. J Cell Physiol. 2019. 10.1002/jcp.28248 30714151

[9] Xiong X , Shi Q , Yang X , Wang W , Tao J . LINC00052 functions as a tumor suppressor through negatively modulating miR‐330‐3p in pancreatic cancer. J Cell Physiol. 2019;234(9):15619‐15626. 30712321 10.1002/jcp.28209

[10] Zhang H , Feng X , Zhang M , et al. Long non‐coding RNA CASC2 upregulates PTEN to suppress pancreatic carcinoma cell metastasis by downregulating miR‐21. Cancer Cell Int. 2019;19:18. 30675129 10.1186/s12935-019-0728-y PMC6335738

[11] Liu JH , Chen G , Dang YW , Li CJ , Luo DZ . Expression and prognostic significance of lncRNA MALAT1 in pancreatic cancer tissues. Asian Pac J Cancer Prev. 2014;15(7):2971‐2977. 24815433 10.7314/apjcp.2014.15.7.2971

[12] Permuth JB , Chen DT , Yoder SJ , et al. Linc‐ing circulating long non‐coding RNAs to the diagnosis and malignant prediction of intraductal papillary mucinous neoplasms of the pancreas. Sci Rep. 2017;7(1):10484. 28874676 10.1038/s41598-017-09754-5 PMC5585319

[13] Jiang S , Tan B , Zhang X . Identification of key lncRNAs in the carcinogenesis and progression of colon adenocarcinoma by co‐expression network analysis. J Cell Biochem. 2019;120(4):6490‐6501. 30430631 10.1002/jcb.27940

[14] Hiraoka N , Yamazaki‐Itoh R , Ino Y , et al. CXCL17 and ICAM2 are associated with a potential anti‐tumor immune response in early intraepithelial stages of human pancreatic carcinogenesis. Gastroenterology. 2011;140(1):310‐321. 20955708 10.1053/j.gastro.2010.10.009

[15] Jury RP , Thibodeau BJ , Fortier LE , et al. Gene expression changes associated with the progression of intraductal papillary mucinous neoplasms. Pancreas. 2012;41(4):611‐618. 22273699 10.1097/MPA.0b013e31823d7b36

[16] Furukawa T , Kloppel G , Volkan Adsay N , et al. Classification of types of intraductal papillary‐mucinous neoplasm of the pancreas: a consensus study. Virchows Arch. 2005;447(5):794‐799. 16088402 10.1007/s00428-005-0039-7

[17] Oldham MC , Langfelder P , Horvath S . Network methods for describing sample relationships in genomic datasets: application to Huntington's disease. BMC Syst Biol. 2012;6:63. 22691535 10.1186/1752-0509-6-63 PMC3441531

[18] Kim D , Langmead B , Salzberg SL . HISAT: a fast spliced aligner with low memory requirements. Nat Methods. 2015;12(4):357‐360. 25751142 10.1038/nmeth.3317 PMC4655817

[19] Williams O , Del Genio CI . Degree correlations in directed scale‐free networks. PLoS ONE. 2014;9(10):e110121. 25310101 10.1371/journal.pone.0110121 PMC4195702

[20] Yip AM , Horvath S . Gene network interconnectedness and the generalized topological overlap measure. BMC Bioinformatics. 2007;8:22. 17250769 10.1186/1471-2105-8-22 PMC1797055

[21] Ravasz E , Somera AL , Mongru DA , Oltvai ZN , Barabasi AL . Hierarchical organization of modularity in metabolic networks. Science. 2002;297(5586):1551‐1555. 12202830 10.1126/science.1073374

[22] Albert R . Scale‐free networks in cell biology. J Cell Sci. 2005;118(Pt 21):4947‐4957. 16254242 10.1242/jcs.02714

[23] Smillie CL , Sirey T , Ponting CP . Complexities of post‐transcriptional regulation and the modeling of ceRNA crosstalk. Crit Rev Biochem Mol Biol. 2018;53(3):231‐245. 29569941 10.1080/10409238.2018.1447542 PMC5935048

[24] Duguang L , Jin H , Xiaowei Q , et al. The involvement of lncRNAs in the development and progression of pancreatic cancer. Cancer Biol Ther. 2017;18(12):927‐936. 29053398 10.1080/15384047.2017.1385682 PMC5718823

[25] McCleary‐Wheeler AL , Lomberk GA , Weiss FU , et al. Insights into the epigenetic mechanisms controlling pancreatic carcinogenesis. Cancer Lett. 2013;328(2):212‐221. 23073473 10.1016/j.canlet.2012.10.005 PMC3513548

[26] Wang KC , Yang YW , Liu B , et al. A long noncoding RNA maintains active chromatin to coordinate homeotic gene expression. Nature. 2011;472(7341):120‐124. 21423168 10.1038/nature09819 PMC3670758

[27] Giulietti M , Righetti A , Principato G , Piva F . LncRNA co‐expression network analysis reveals novel biomarkers for pancreatic cancer. Carcinogenesis. 2018;39(8):1016‐1025. 29796634 10.1093/carcin/bgy069

[28] Fu XL , Liu DJ , Yan TT , et al. Analysis of long non‐coding RNA expression profiles in pancreatic ductal adenocarcinoma. Sci Rep. 2016;6:33535. 27628540 10.1038/srep33535 PMC5024322

[29] Yan Y , Li S , Wang S , et al. Long noncoding RNA HAND2‐AS1 inhibits cancer cell proliferation, migration, and invasion in esophagus squamous cell carcinoma by regulating microRNA‐21. J Cell Biochem. 2019;120(6):9564‐9571. 30520131 10.1002/jcb.28233

[30] Miao F , Chen J , Shi M , Song Y , Chen Z , Pang L . LncRNA HAND2‐AS1 inhibits non‐small cell lung cancer migration, invasion and maintains cell stemness through the interactions with TGF‐beta1. Biosci Rep. 2019;39(1). 10.1042/BSR20181525 PMC6328884 30509963

[31] Zhou J , Lin J , Zhang H , Zhu F , Xie R . LncRNA HAND2‐AS1 sponging miR‐1275 suppresses colorectal cancer progression by upregulating KLF14. Biochem Biophys Res Comm. 2018;503(3):1848‐1853. 30078677 10.1016/j.bbrc.2018.07.125

[32] Kang Y , Zhu X , Xu Y , et al. Energy stress‐induced lncRNA HAND2‐AS1 represses HIF1alpha‐mediated energy metabolism and inhibits osteosarcoma progression. Am J Cancer Res. 2018;8(3):526‐537. 29637006 PMC5883101

[33] Yang X , Wang CC , Lee WYW , Trovik J , Chung TKH , Kwong J . Long non‐coding RNA HAND2‐AS1 inhibits invasion and metastasis in endometrioid endometrial carcinoma through inactivating neuromedin U. Cancer Lett. 2018;413:23‐34. 29107108 10.1016/j.canlet.2017.10.028

